# Primary Extragastrointestinal Stromal Tumor of the Dorsolateral Anorectum Resected via a Posterior Approach: A Case Report

**DOI:** 10.70352/scrj.cr.25-0548

**Published:** 2025-12-05

**Authors:** Makoto Hasegawa, Wataru Sakamoto, Norshalena Shakespear, Hiroki Yago, Takahiro Sato, Misato Ito, Takuro Matsumoto, Daisuke Ujiie, Shun Chida, Hirokazu Okayama, Motonobu Saito, Tomoyuki Momma, Koji Kono

**Affiliations:** 1 Department of Gastrointestinal Tract Surgery, Fukushima Medical University School of Medicine, Fukushima, Fukushima, Japan; 2 Department of Diagnostic Pathology, Fukushima Medical University School of Medicine, Fukushima, Fukushima, Japan

**Keywords:** extra-gastrointestinal GIST, posterior approach, rectal GIST

## Abstract

**INTRODUCTION:**

Gastrointestinal stromal tumors (GISTs) can arise anywhere along the gastrointestinal tract. When they originate outside the gastrointestinal tract, they are referred to as extragastrointestinal stromal tumors (EGISTs), which are rare. In particular, EGISTs arising in the perianorectal region are extremely rare. We report the case of a primary perianorectal EGIST, which was initially diagnosed preoperatively as a rectal GIST.

**CASE PRESENTATION:**

A 74-year-old woman presented with a palpable mass on her left buttock. Histopathological analysis of the biopsy specimen confirmed the diagnosis of GIST. MRI revealed a 46 × 28 mm mass on the left dorsolateral aspect of the lower rectum, which was preoperatively diagnosed as rectal GIST. Partial resection of the rectum via a posterior approach was planned. Intraoperatively, however, no continuity between the tumor and rectal wall was observed. Based on pathological and operative findings, the tumor was diagnosed as a perianorectal EGIST, and complete resection was achieved without rectal resection.

**CONCLUSIONS:**

This case underscores the clinical significance of considering EGIST as a differential diagnosis for anorectal GIST lacking clear continuity with the intestinal wall. Furthermore, our experience suggests that posterior approach is less invasive, facilitates anus preservation, and should be considered for resection of both rectal GISTs and perianorectal EGISTs.

## INTRODUCTION

Gastrointestinal stromal tumors (GISTs) are the most prevalent mesenchymal neoplasms of the gastrointestinal (GI) tract, originating from the interstitial cells of Cajal (ICCs), which function as the pacemaker cells regulating GI peristalsis through mediation between autonomic nervous inputs and smooth muscle contraction.^1)^ Typically, GISTs present as subepithelial masses, correlating anatomically with ICC distribution. GISTs occur anywhere along the GI tract; they are most common in the stomach (50%–60%) and small intestine (30%–35%), they may also occur, although less frequently, in the colon, rectum (approximately 5%), and esophagus (<1%).^1)^ Rarely, GISTs may be found in sites outside the gastrointestinal tract, called extragastrointestinal stromal tumors (EGISTs). EGISTs often occur in the omentum, mesentery, or retroperitoneum; among EGISTs, those arising in the perianorectal region are extremely rare, with only 2 cases previously reported.^1–5)^

We herein report a rare case of primary EGIST located on the left dorsolateral aspect of the lower rectum, which was initially misdiagnosed as a rectal GIST based on preoperative imaging. Surgical resection was performed via a posterior approach; however, intraoperative findings revealed no anatomical continuity with the rectum, leading to the final diagnosis of a primary extragastrointestinal stromal tumor on the left dorsolateral aspect of the lower rectum.

## CASE PRESENTATION

**Fig. 1:**
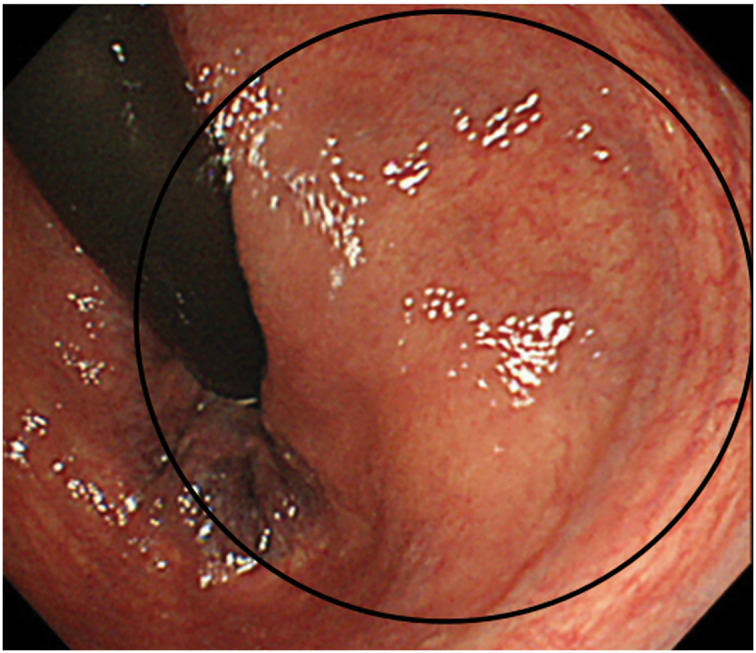
The image of the colonoscopy, which revealed external compression of the posterior wall of the rectum.

**Fig. 2:**
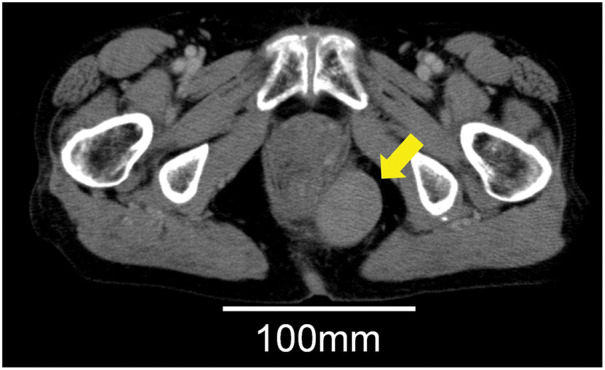
Contrast-enhanced CT (CECT) revealed a 46 × 28 mm mass on the left dorsolateral aspect of the lower rectum (yellow arrow).

**Fig. 3:**
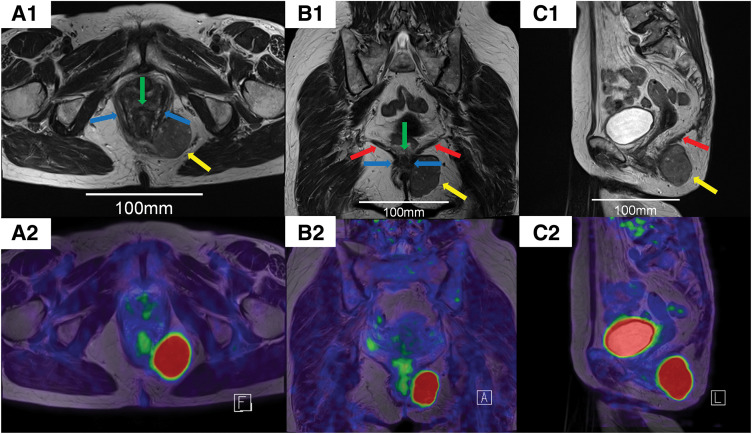
MRI and fused PET-MRI of the tumor. T2-weighted MRI scans are shown in the (**A1**) axial, (**B1**) coronal, and (**C1**) sagittal planes. Corresponding fused PET-MRI images are shown in the (**A2**) axial, (**B2**) coronal, and (**C2**) sagittal planes. A well-defined 46 × 28 mm mass is noted on the left dorsolateral aspect of the lower rectum, which is T1-hypointense, T2-hyperintense, and demonstrates intense fluorodeoxyglucose accumulation, which was in contact with the puborectal muscle. The continuity with the rectum was unclear. The tumor is indicated by the yellow arrow, the rectum by the green arrow, the puborectal muscle by the blue arrows, and the levator ani muscle by the red arrows.

A 74-year-old woman presented with a palpable mass on her left buttock, which she first noticed approximately 7 months prior. Ultrasonography performed at a previous hospital revealed a hypoechoic mass in the left buttock. Histopathological analysis of the biopsy specimen confirmed the diagnosis of GIST, and the patient was referred to our institution for further evaluation and treatment. Colonoscopy revealed external compression of the posterior wall of the rectum (**Fig. 1**). Contrast-enhanced CT (CECT) revealed a 46 × 28 mm mass on the left dorsolateral aspect of the lower rectum (**Fig. 2**). MRI revealed a 46 × 28 mm mass on the left dorsolateral aspect of the lower rectum (**Fig. 3**). The mass was T1-hypointense, T2-hyperintense, and showed no signal suppression on fat-suppressed sequences. It was found to be in contact with the puborectal muscle, and there was no clear continuity with the rectal wall (**Fig. 3**). PET showed fluorodeoxyglucose accumulation within the mass (**Fig. 3**). No other lesions were noted on upper gastrointestinal endoscopy and CECT.

**Fig. 4:**
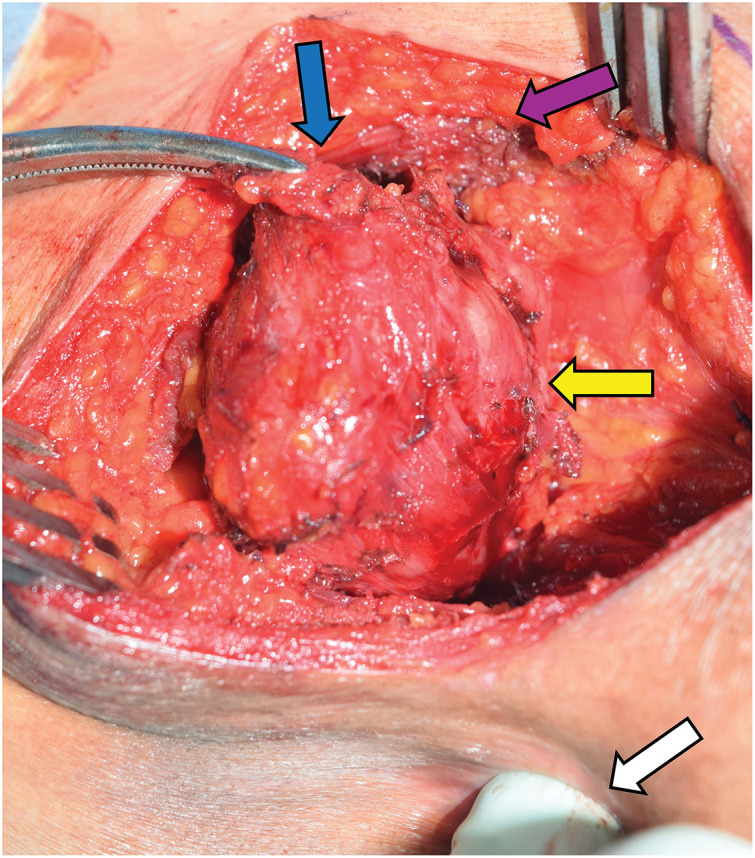
Intraoperative image. The absence of continuity between the tumor and the rectum was confirmed by digital rectal examination. The tumor is indicated by the yellow arrow, and the surgeon’s finger performing the examination is indicated by the white arrow. Part of the puborectal muscle was adherent to the tumor (blue arrow). Part of the gluteus maximus muscle was separated (violet arrow).

A partial resection of the rectum via a posterior approach was planned under the preoperative diagnosis of rectal GIST. The procedure was initiated at jackknife position. A 10-cm skin incision was made 3 cm from the anal verge and 1 cm lateral to the left border of the sacrum. The subcutaneous fat and part of the gluteus maximus muscle were separated. The tumor was identified on the outer aspect of the puborectal muscle. A circumferential dissection of the tumor was performed, and an ultrasonic coagulating incision device was employed to achieve hemostasis. This was necessary due to the tumor’s vascularity. Part of the puborectal muscle was adherent to the tumor, then part of the puborectal muscle was incised for complete resection. The absence of continuity between the tumor and the rectum was confirmed intraoperatively by digital rectal examination (**Fig. 4**). Complete resection of the tumor was achieved without requiring partial resection of the rectum. No tumor rupture was identified. After tumor removal, the surgical field was irrigated with sterile saline. The dissected portions of the puborectal muscle and gluteus maximus muscle were repaired, and the wound was subsequently closed to complete the procedure. The operative time was 76 min, and intraoperative blood loss was 50 mL.

**Fig. 5:**
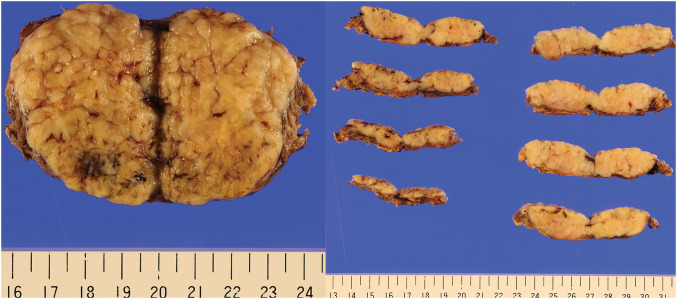
Macroscopic appearance, resected tumor cut surface (measured 55 × 40 mm): Well-circumscribed solid tumor showing pale yellow cut surface, with neither signs of necrosis nor hemorrhage.

**Fig. 6:**
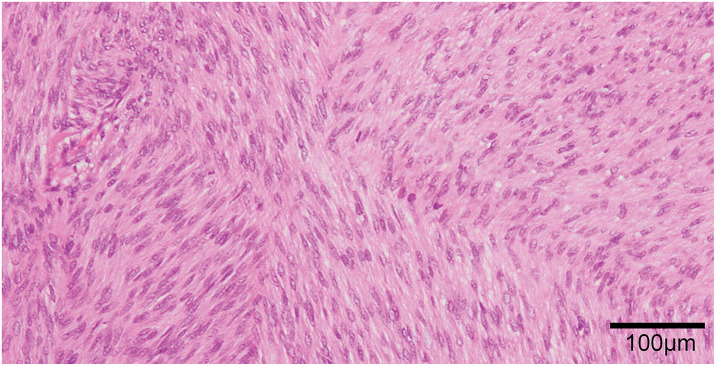
Hematoxylin and eosin staining (100× magnification): Tumor consisted of interlacing fascicles of spindle tumor cells with elongated nuclei and eosinophilic cytoplasm.

**Fig. 7:**
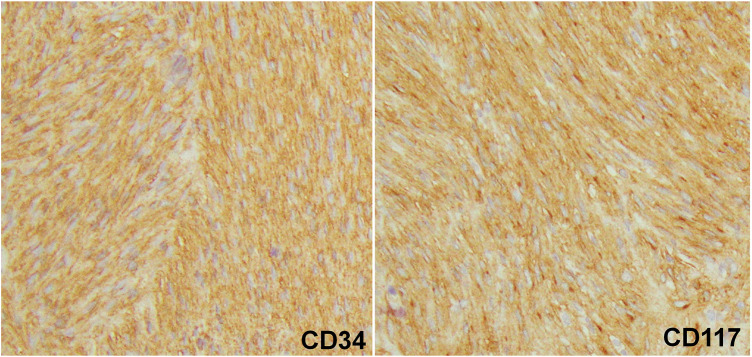
Immunohistochemical staining (100× magnification): Spindle tumor cells stained positive for CD34 and CD117(c-kit).

The resected tumor measured 55 × 40 mm (**Fig. 5**). Histology showed a well-circumscribed spindle cell tumor (**Fig. 6**). Immunohistochemical staining revealed positivity for vimentin, CD34, CD117, and DOG1, and negativity for AE1/3, desmin, SMA, S100P, and STAT6. The Ki-67 labeling index was 3.5% (**Fig. 7**). The mitotic count was 2 per 10 high-power fields (HPFs). Genetic analysis was not performed. Based on the pathological and operative findings, the tumor was diagnosed as an EGIST. Pathological examination confirmed complete resection with negative margins. The patient was judged to be at high risk for recurrence according to the modified-Fletcher classification, based on the tumor size (55 mm), mitotic rate (equivalent to 10 per 50 HPF), and non-gastric primary site.^6,7)^ Since the patient was judged to be high-risk according to the modified-Fletcher classification, the plan was to administer adjuvant therapy with oral imatinib for 3 years postoperatively.

The patient resumed oral intake on POD 1 and was discharged on POD 4 without complications. At the initial outpatient assessment, the patient reported no fecal incontinence, with a Wexner score of 0. Adjuvant therapy with imatinib was initiated; however, it was discontinued after 3 months due to an allergic reaction. At the 7-month follow-up, there was no evidence of recurrence.

## DISCUSSION

We identified 2 clinically important observations based on this case. First, although rare, GISTs located in the perianorectal region may lack anatomical continuity with the anorectum, thus rectal resection may not be necessary in such cases. Second, regardless of the presence or absence of anorectal continuity, the posterior approach may offer an effective and minimally invasive route to achieve complete resection of both anorectal GIST and perianorectal extragastrointestinal GISTs.

In the present case, the tumor was preoperatively misdiagnosed as a rectal GIST due to its anatomical location beside the anorectum, a misdiagnosis likely compounded by the extreme rarity of EGIST in this region. However, intraoperative findings revealed no continuity with the intestinal wall, leading to a final diagnosis of EGIST arising in the buttock, emphasizing the clinical importance of thorough intraoperative assessment. While GISTs predominantly occur within the GI tract, EGISTs are uncommon, and those arising outside the abdominal cavity are exceedingly rare. To date, only a limited number of EGISTs originating in the vicinity of the anorectum have been reported. Kang et al. described a perianal EGIST located at the 11 o’clock position in the perianal area,^4)^ while Elagami et al. reported a perirectal EGIST situated anterior to the rectum and posterior to the bladder.^5)^ Hence, in anorectal GIST without clear intestinal continuity, clinicians must consider EGIST as a differential diagnosis (**Table 1**).

**Table 1:** Clinicopathological features of reported cases of perianorectal extragastrointestinal stromal tumors.

Author	Year	Age (years old)	Sex	Location	Size (mm)	Rectal continuity	Procedure	Margin status	Complications	Mitoses (HPF)	Ki-67	KIT mutation	PDGFRA mutation	Adjuvant therapy	Follow-up (months)	Outcome
Kang et al.^4)^	2020	70	Male	Anterior lateral perianal region (11 o’clock position in the perianal area)	43 × 32	No	Local excision	R0	None	1/50	<1.0%	Presence	N/A	N/A	6	Recurrence-free survival
Elagami et al.^5)^	2021	41	Male	The posterior dome of the bladder (anterior to the rectum)	70	No	Open transabdominal resection	N/A	None	5/50	<5.0%	Presence	absence	Imatinib	4	Recurrence-free survival
Present case	–	74	Female	Left dorsolateral aspect of the lower rectum	55 × 40	No	Posterior approach	R0	None	2/10	3.5%	N/A	N/A	Imatinib	7	Recurrence-free survival

HPF, high-power fields; N/A, not available; R0, complete resection with negative margins

The precise etiology of EGIST remains uncertain. It has been proposed that some EGISTs may represent metastases from undetected primary GISTs, particularly when occurring within the abdominal cavity, such as in the omentum, mesentery, or retroperitoneum.^1,8)^ In contrast, EGISTs arising in the perianorectal region are exceedingly rare.^1)^ Given that GIST metastases typically involve intra-abdominal sites—such as the liver, omentum, and peritoneum—while extra-abdominal metastasizes are uncommon, EGISTs located outside the abdominal cavity, such as in our case, may be more likely to represent true primary tumors rather than secondary lesions.

In this case, a less invasive surgical approach was feasible. Had this tumor been managed as a rectal GIST without employing the posterior approach, complete resection would likely have necessitated an abdominoperineal resection, thereby resulting in permanent colostomy. In contrast, the posterior approach might enable complete tumor resection without partial rectal resection. Even in cases of rectal GIST, the posterior approach may facilitate anus preservation and reduce the risk of postoperative low anterior resection syndrome.^9)^ However, anorectal EGIST is very rare, and the accumulation of more case reports is necessary to establish the optimal surgical approach.

In this case, the patient was classified as being at high risk of recurrence, and adjuvant chemotherapy with imatinib for 3 years was planned following surgical resection. According to Japanese Clinical Practice Guidelines 2022 for GIST issued by the Japan Society of Clinical Oncology,^10)^ recurrence risk should be evaluated using the Modified Fletcher/Joensuu classification systems,^6,7)^ and adjuvant imatinib therapy for 3 years is recommended for high recurrence risk cases or those with tumor rupture. Beyond these classifications, the prognosis of EGISTs arising outside the GI tract is generally considered poorer; however, due to the rarity of these tumors, the benefits of adjuvant chemotherapy are not well established.^8)^

## CONCLUSIONS

We report a rare case of primary EGIST located on the left dorsolateral aspect of the lower rectum, initially diagnosed preoperatively as rectal GIST. This case underscores the clinical significance of considering EGIST as a differential diagnosis for anorectal GIST lacking clear continuity with the intestinal wall. Furthermore, our experience suggests that the posterior approach might be a feasible, anus-preserving surgical option, warranting consideration for selected cases of both rectal GIST and perianorectal EGIST.
